# Rapid severe hypertension and organ damage in two‐kidney two‐clip rats produced by different sizes of clips

**DOI:** 10.1002/ame2.70027

**Published:** 2025-05-14

**Authors:** Jia‐Sheng Tian, Qi‐Sheng Ling, Yu‐Chen Wei, Dao‐Xin Wang, Chao‐Yu Miao

**Affiliations:** ^1^ Department of Pharmacology Second Military Medical University/Naval Medical University Shanghai China; ^2^ School of Medicine Shanghai University Shanghai China

**Keywords:** hypertension, organ damage, renal artery stenosis, renin‐angiotensin‐aldosterone system, two‐kidney two‐clip

## Abstract

**Background:**

The two‐kidney two‐clip (2K2C) rat model is widely used in hypertension, stroke, and drug studies. However, knowledge of this model is quite limited.

**Methods:**

In this study, U‐shaped silver clips with inner diameters of 0.3, 0.25, and 0.2 mm were used to narrow bilateral renal arteries, inducing different extents of blood flow reduction for preparing 2K2C rats. Blood pressure (BP) was continuously measured during 2K2C procedures until 10 h after 2K2C, and long‐term assessments of BP, organ damage, and serum biochemistry were performed at 1, 2, 4, and 6 weeks.

**Results:**

BP increased immediately when the 2K2C rats regained consciousness from anesthesia, with a 100% incidence of hypertension on the day of operation, and increased to a plateau at 2 weeks for 0.2 mm clips (248 mmHg), 4 weeks for 0.25 mm clips (219 mmHg), and perhaps 6 weeks for 0.3 mm clips (206 mmHg). BP levels displayed a negative correlation with clip sizes. Organ damage, including aortic and cardiac hypertrophy, cerebrovascular and cardiovascular injury, and renal atrophy or compensatory enlargement, occurred as early as 1 or 2 weeks after 2K2C. Routine serum biochemistry indicated organ dysfunction primarily at 4–6 weeks in 0.2 mm clips, whereas aspartate aminotransferase appeared a sensitive biomarker for early organ damage.

**Conclusion:**

2K2C can produce severe hypertension and multi‐organ damage in a short time. 2K2C rats in 0.2 mm clips can be used as a hypertensive emergency model. This study provides crucial insights for guiding the development of targeted interventions.

## Graphical abstract

Blood pressure increases immediately when the 2K2C rats (made with 0.3, 0.25, and 0.2 mm U‐shaped clips) regain consciousness from anesthesia. 100% of the rats develop hypertension on the day of 2K2C surgery. Blood pressure continues to rise, resulting in severe hypertension and organ damage to the brain, heart, aorta, and kidneys at 1, 2, 4, and 6 weeks after 2K2C surgery. The 2K2C rat model made with 0.2 mm clips is also suitable for hypertensive emergency.

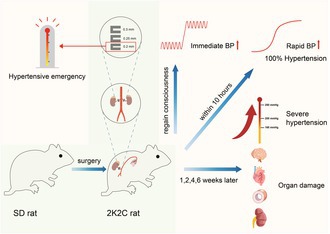

## 1 INTRODUCTION

Hypertension is a major risk factor for cardiovascular and cerebrovascular events and is one of the World Health Organization (WHO) global targets for prevention.^1^, ^2^, ^3^, ^4^ Renal artery stenosis is a common secondary cause of hypertension^5^ and affects 1%–10% of hypertension patients.^1^, ^2^, ^3^, ^6^ Decreased renal perfusion leads to the release of renin and in turn activates the renin‐angiotensin‐aldosterone system (RAAS), which promotes hypertension and triggers a cascade of events.^7^, ^8^, ^9^

Renal artery stenosis can be categorized into unilateral and bilateral types, corresponding to renovascular hypertensive rats known as two‐kidney one‐clip (2K1C) and two‐kidney two‐clip (2K2C), respectively.^10^, ^11^, ^12^ Bilateral renal artery stenosis usually causes more severe hypertension and organ damage compared to the unilateral renal artery stenosis, and the 2K2C rats are also frequently used as a stroke‐prone renovascular hypertension model.^11^, ^13^ Previous rat studies had demonstrated the 100% incidence of hypertension at 2 weeks after 2K2C surgery,^11^ and the systolic BP could reach up to 250 mmHg, with obvious damages to the brain, heart, aorta, and kidneys.^12^

Although the 2K2C rat model has been used in numerous research studies, we are still curious about its similarity with the hypertension‐related syndromes in clinical settings. For example, when does the animal's BP rise? In addition, the clip size of 2K2C model is not uniform; most studies use 0.3 mm clips, whereas some use 0.25 or 0.2 mm.^11^, ^13^, ^14^, ^15^, ^16^ There is no accurate understanding of the relationship between BP and the degree of renal artery stenosis in this model. Therefore, we try to know whether there exist significant differences in BP, organ damage, or blood biochemistry among the 2K2C rats with different stenosis degrees. An in‐depth study is necessary. We thus used different‐sized U‐shaped silver clips (with inner diameters of 0.3, 0.25, and 0.2 mm) to narrow the bilateral renal arteries in rats, assessed the changes in BP at different times during and after the 2K2C surgery, and measured the organ morphological and functional impairments to describe more about this model.

## 2 MATERIALS AND METHODS

### 2.1 Animals and preparation of 2K2C rats

Animal experiments were approved by the Committee on Ethics of Medical Research, Second Military Medical University/Naval Medical University, and performed in adherence with the National Institute of Health Guide for the Care and Use of Laboratory Animals. Male Sprague–Dawley (SD) rats (140–160 g) were purchased from Sino‐British SIPPR/BK Lab Animal Ltd. (Shanghai, China), and 148 rats were used. All rats were housed in standardized individually ventilated cages (IVC, temperature 23–25°C, humidity 40%–60%), were under lighting 8:00–20:00, and received standard animal chow and tap water ad libitum.

2K2C surgery was performed using different‐sized U‐shaped silver clips (0.3, 0.25, and 0.2 mm, Alcott Biotech Co., Ltd., Shanghai, China). As we described previously,^11^, ^12^ rats were made to fast for 12 h (20:00–08:00) but had free access to drinking water before surgery to improve the surgical visual field. Then rats were anesthetized by intraperitoneal injection of 2% pentobarbital sodium (40 mg/kg, Sigma‐Aldrich, Shanghai, China) and tied to a platform; their abdomen was shaved and sterilized, and a 1.5‐cm‐long incision was made 1.5 cm below the xiphoid process. The incision was enlarged with a spreader, and the intestines were then moved aside with a sterilized swab to fully expose the left kidney. The kidney was rolled over, the renal artery was exposed and isolated with micro‐forceps, and then a silver clip was placed (0.3, 0.25, or 0.2 mm) to narrow the renal artery. The same procedures were performed on the right renal artery. The kidneys and intestines were repositioned, penicillin (Xinxiang Huachu Trading Co., Ltd., Xinxiang, China) was administered for anti‐infection, and then the skin was sutured. The same procedure without the placement of clips was performed for sham‐operated rats as sham controls. After the surgery, the rats continued to fast for 24 h, which helped reduce the death in the acute phase.

### 2.2 Measurement of blood flow after renal artery stenosis

Rats were anesthetized with 3% isoflurane gas (RWD Life Science Co., LTD., Shenzhen, China), and maintained at 2% concentration. The right renal artery was exposed in the same way as in the 2K2C surgery, and a 0.5 mm ultrasound blood flow probe was placed around the artery,^17^ which was combined with a Transonic blood flow meter and PowerLab data acquisition system (AD Instruments International Trading Co., LTD., Shanghai, China). The renal blood flow was recorded for 5 min as a basal level, then a 0.3, 0.25, or 0.2 mm silver clip was placed between the probe and abdominal aorta to lower blood flow, and the renal blood flow was recorded for another 5 min to calculate the reduction. 28 rats were used in this part.

### 2.3 BP and heart rate measurement for acute and long‐term observation

**FIGURE 1:**
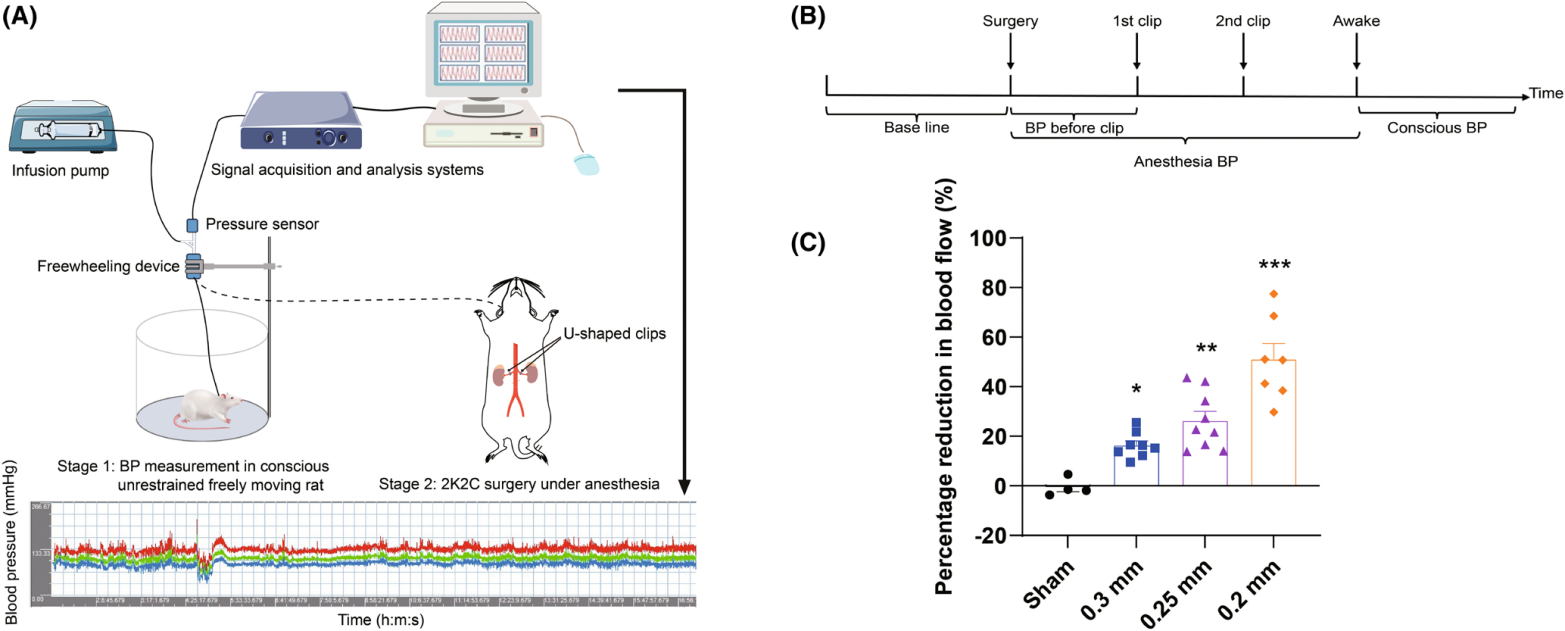
Blood pressure (BP) measurement and renal artery blood flow changes in rats. (A) Experimental schematic diagram for BP measurement during two‐kidney two‐clip (2K2C) surgery. (B) Experimental protocol for BP measurement. (C) Reduction in renal artery blood flow by three different sizes of silver clips with inner diameters of 0.3, 0.25, and 0.2 mm, respectively. Data are shown as the mean ± standard error mean (SEM). *n* = 4–9 animals per group. *p < 0.05, **p < 0.01, ***p < 0.001 versus sham. One‐way analysis of variance (ANOVA) followed by Dunnett's test is used.

Baseline BP and heart rate measurement, that is, measurement in conscious unrestrained freely moving rats, was performed as we described previously.^11^, ^12^ Briefly, rats were anesthetized with 2% pentobarbital sodium (40 mg/kg) by intraperitoneal injection. Then they were placed in a supine position, and the left femoral arteries were isolated. A polyethylene catheter (PE10 tubing: inner diameter 0.28 mm, outer diameter 0.61 mm; PE50 tubing: inner diameter 0.58 mm, outer diameter 0.96 mm, joined and fused) filled with sodium heparin (50 U/L, Shanghai Boguang Biotech Co., Ltd., Shanghai, China) was inserted into the abdominal aorta via a femoral artery incision to measure BP and heart rate. The other end of the catheter was exteriorized through the interscapular skin, and fixed with a special saddle. Twenty‐four hours later, each rat was placed in a cylindrical cage with food and water. The catheter was then connected to a BP transducer via a rotating spindle that allowed the rat to move freely, then the BP was synchronously recorded by an MPA‐HBBS data acquisition and analysis system (Alcott Biotech Co., Ltd.). Sodium heparin (50 U/L) was infused at 0.3 mL/h into the aortic catheter to prevent coagulation. After 3–4 h of habituation, BP and heart rate were recorded continuously for 2 h (Figure 1A, stage 1).

The acute phase measurements (13 rats were used) were divided into six steps. Step 1. Normal SD rats were anesthetized with 2% pentobarbital sodium, the femoral artery intubation was performed, and the catheter was drawn from the neck and fixed for BP measurement. Twenty‐four hours later, the rats were subjected to conscious unrestrained freely moving BP measurements (using an extended catheter), which were used to record baselines. Step 2. After that, the rats were taken out and anesthetized with 3% isoflurane (maintained with 2% isoflurane), and the bilateral renal arteries were isolated, then the abdominal cavity was simply closed and the BP of anesthetized rats was recorded (Figure 1A, stage 2). Step 3. The abdominal cavity was reopened; a 0.3, 0.25, or 0.2 mm clip was placed in one renal artery, and recorded stable BP for 5 min, and then another clip was placed on the opposite renal artery and recorded for another 5 min. Step 4. Penicillin was then administered, and the skin was quickly sutured. Step 5. After the isoflurane gas was stopped, the rats were moved back to the cages, and after 1–2 min, the rats became awake. Step 6. BP was continuously recorded during the above procedures and during 10 h after the procedures (protocol shown in Figure 1B).

For long‐term observation (we did it twice, 36 and 71 rats were used, respectively), a new batch of 2K2C and sham rats were prepared. The body weight and food intake were measured before and after 2K2C surgery; briefly, the rats were placed in a single cage when measured, and the food consumption of each rat was recorded for 24 h. At 1, 2, 4, and 6 weeks after surgery, several rats were randomly selected from each group for BP and heart rate measurements in conscious unrestrained freely moving state, as described above. BP was measured once for each rat, and after BP measurement the rats were killed for evaluation of organ morphology and/or serum biochemistry.

### 2.4 Morphological analysis

After the BP and heart rate measurement at 1, 2, 4, and 6 weeks after 2K2C surgery or sham operation (also twice), rats were anesthetized with 2% pentobarbital sodium (40 mg/kg). Then they were perfused with pre‐cooled saline at 4°C to flush blood from the tissues. The brain, heart, aorta, liver, and bilateral kidneys were removed for measurements of organ weight and others.^18^, ^19^, ^20^, ^21^ The aorta was cleaned of adhering fat and connective tissue before it was removed, and then cut from the left subclavian artery branch to the diaphragm. After the aorta was removed, it was slightly cleaned, dried, and weighed. The aortic length was measured, and the aortic weight to length ratio was calculated.^11^, ^20^ The whole heart was removed, cleaned, drained, and weighed. The different parts of the heart were measured according to the following steps. Step 1. The atria were cut off to obtain the whole ventricle, and the ventricle was weighed. Step 2. The right ventricle was cut to obtain the whole left ventricle, and the left ventricle was weighed. Step 3. The left ventricle was cut across in the same position (middle), and the left ventricular free wall thickness was measured using a Vernier caliper at three different locations (mastoid muscle not included), and the average value was taken. The kidneys were weighed. Each kidney was divided into two flaps, and the boundary between cortex and medulla could be directly seen. We first measured the renal thickness from the renal pedicle to the edge, then measured the thickness of the cortex, and the medullary thickness was equal to the renal thickness minus cortical thickness. We measured three different positions at the top, middle, and bottom of kidney, and the average value was taken.

Thereafter, for histological analysis, the brain, heart, aorta, liver, and kidneys were fixed with 4% paraformaldehyde, paraffin‐embedded, and sliced (4 μm thickness), and then used for various staining methods, including Verhoeff's Van Gieson (EVG),^22^ hematoxylin–eosin (H&E), and Masson's trichrome staining. We quantified the scoring of bilateral cerebrovascular lesions by EVG staining^11^, ^23^; and H&E staining for liver injury, myocardial hypertrophy, aortic thickness measurements and renal damage; Masson staining for collagen content in cardiac and renal fibrosis.^24^, ^25^ Software Image‐Pro Plus version 6.0 was used.

### 2.5 Serum biochemical assays

At 1, 2, 4, and 6 weeks after 2K2C surgery or sham operation, rats were anesthetized with 3% isoflurane gas, and orbital blood (approximately 1 mL) was collected from each rat.^26^ Then the blood was centrifuged at 3000*g* for 10 min, and serum was collected. Serum levels of creatine kinase (CK), lactate dehydrogenase (LDH), alanine aminotransferase (ALT), aspartate aminotransferase (AST), urea, creatinine, uric acid, Na^+^, and K^+^ were measured using an automatic biochemical analyzer (Hitachi High Technology Co., LTD., Nagako, Japan).

### 2.6 Statistical analysis

All experimental data are expressed as mean ± standard error of mean (SEM). Statistical analysis was performed with GraphPad Prism 10 software (GraphPad, San Diego, CA, USA). The data were tested for normality by Kolmogorov–Smirnov test or Shapiro–Wilk test. For normally distributed data, statistical differences were determined using one‐way analysis of variance (ANOVA) or two‐way ANOVA followed by Dunnett's test for comparisons among multiple groups, or using a paired t test for comparisons with its baseline in each group. For non‐normally distributed data, they are normalized by log_10_. Chi‐square test was used for renal atrophy or compensatory enlargement rates. A *p* value <0.05 indicated a statistically significant difference.

## 3 RESULTS

### 3.1 Renal artery stenosis by different sizes of clips in rats reduces renal blood flow in a size‐dependent manner

U‐shaped silver clips of 0.3, 0.25, and 0.2 mm were used to narrow the unilateral renal artery separately. As shown in Figure 1C, blood flow did not change in sham‐operated rats, whereas the 0.3 mm silver clip reduced the blood flow by 16.4%, 0.25 mm by 26.2%, and 0.2 mm by 51.0%. All three silver clip sizes were able to significantly reduce the renal perfusion blood flow, and the reduction was more severe with the decreasing size of the silver clips.

### 3.2 BP increases as soon as rats regain consciousness after 2K2C surgery with a 100% incidence of hypertension on the day of operation

We primarily examined the changes in BP in rats throughout the entire 2K2C surgery and 10 h after the procedure, and we aimed to observe whether the placement of the silver clips immediately elevated the BP.

**FIGURE 2:**
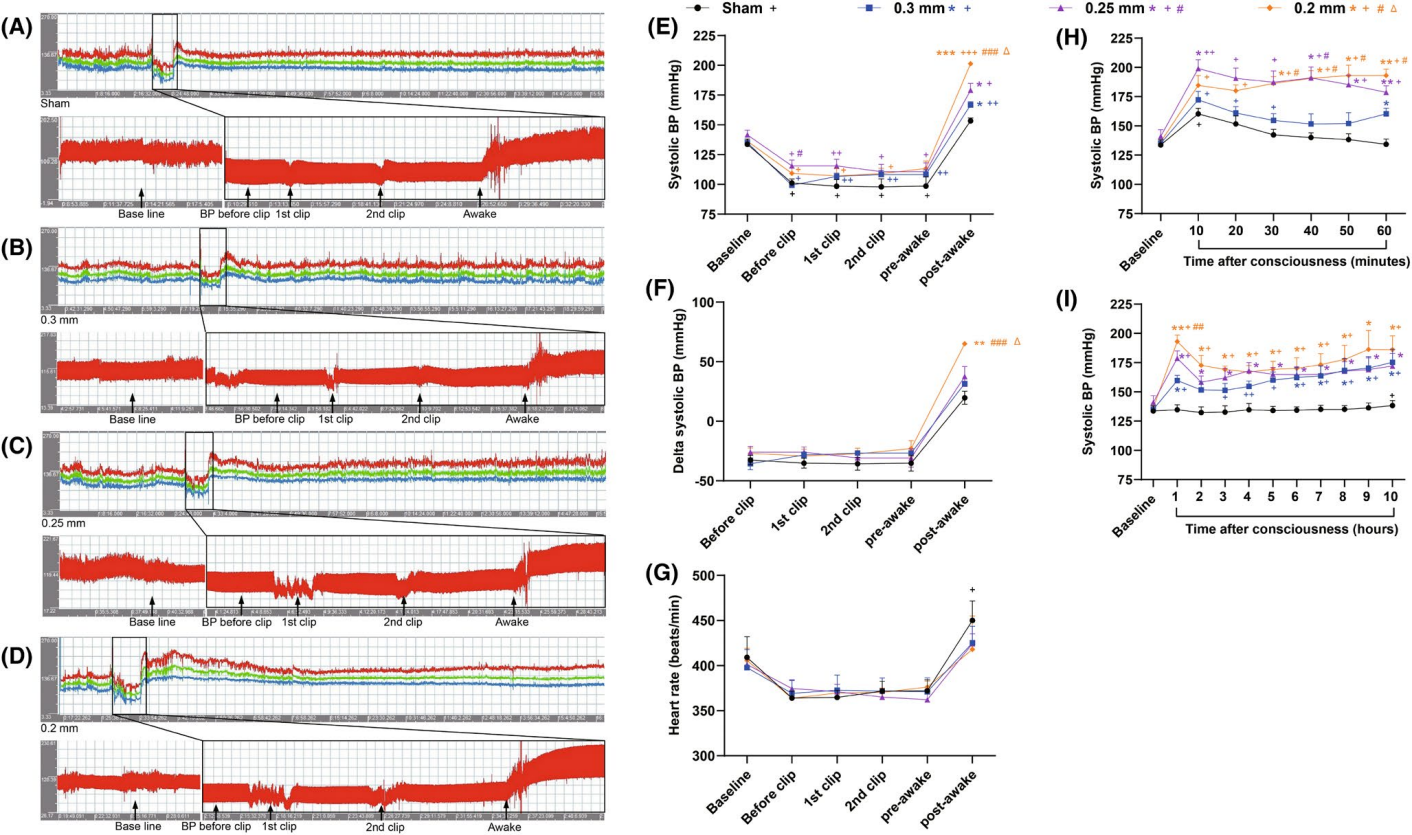
Changes in blood pressure (BP) before, during, and after 2K2C surgery. (A–D) Representative recordings of BP changes and their local magnifications with sham operation (A), 0.3 mm clips (B), 0.25 mm clips (C), and 0.2 mm clips (D) before, during, and after 2K2C surgery. (E–G) Systolic BP (E), delta systolic BP (F), and heart rate (G) before, during, and after 2K2C surgery. (H) Changes in systolic BP within 1 h after consciousness. (I) Changes in systolic BP within 10 h after consciousness. Data are shown as the mean ± standard error mean (SEM). *n* = 3–4 animals per group. *p < 0.05, **p < 0.01, ***p < 0.001 versus sham. ^#^ *p* < 0.05, ^##^ *p* < 0.01, ^###^ *p* < 0.001 versus 0.3 mm. ^Δ^ *p* < 0.05 versus 0.25 mm. Two‐way analysis of variance (ANOVA) followed by Dunnett's test is used. ^+^ *p* < 0.05, ^++^ *p* < 0.01, ^+++^ *p* < 0.001 versus baseline. Paired *t* test is used.

Figure 2A–D shows the representative recordings of BP changes in each group, and Figure 2E–G demonstrates the systolic BP, delta systolic BP, and heart rate levels at each stage, corresponding to the protocol and representative recordings. We first observed that the normal systolic BP in conscious unrestrained freely moving rats was about 140 mmHg, and after anesthesia with isoflurane gas, the BP of each group significantly decreased by an average of about 30 mmHg, compared with its baseline, suggesting that anesthesia results in a BP reduction (Figure 2E).

Then, we prepared the rats for 2K2C procedure, and recorded BP and heart rate signals simultaneously. Results showed that systolic BP in each group did not change before clips were placed, after the first and second clips were placed, and before the rats were awake. However, when the rats became conscious, the systolic BP increased immediately. The 0.3, 0.25, and 0.2 mm groups showed a significant increase in systolic BP and delta systolic BP (only 0.2 mm significant) post‐awake but no effect on heart rate when compared with the sham‐operated rats (Figure 2E–G). When compared with the 0.3 or 0.25 mm groups, the rats in 0.2 mm group showed a significantly higher systolic BP when the rats were awake. The above results indicate that the BP does not increase during the 2K2C surgery under anesthesia but increases immediately once the rats are conscious after the withdrawal of gas anesthesia.

We continued to record BP for the subsequent 10 h. As shown in Figure 2H,I, systolic BP increased rapidly in sham‐operated rats within 10 min after consciousness, and gradually decreased and returned to the baseline within 1 h. It remained stable for the following 9 h. Rats in the 0.3, 0.25, and 0.2 mm groups also had an elevation of systolic BP within 10 min after consciousness. Subsequently, the BP began to decrease and fluctuate. One hour later, the three groups had increased BPs to 160, 179, and 193 mmHg, respectively, with significant differences compared to 135 mmHg in the sham group. BP was measured continuously from 1 to 10 h, and the rats again experienced fluctuations and ultimately were stable, with systolic BPs of 175, 173, and 186 mmHg at the 10th h, respectively. These results suggest that the BP increases to high levels during the acute phase after 2K2C surgery. Hypertension occurs in all rats on the first day after 2K2C operation.

### 3.3 Hypertension reaches a very high plateau at the earliest 2 weeks after 2K2C surgery

**FIGURE 3:**
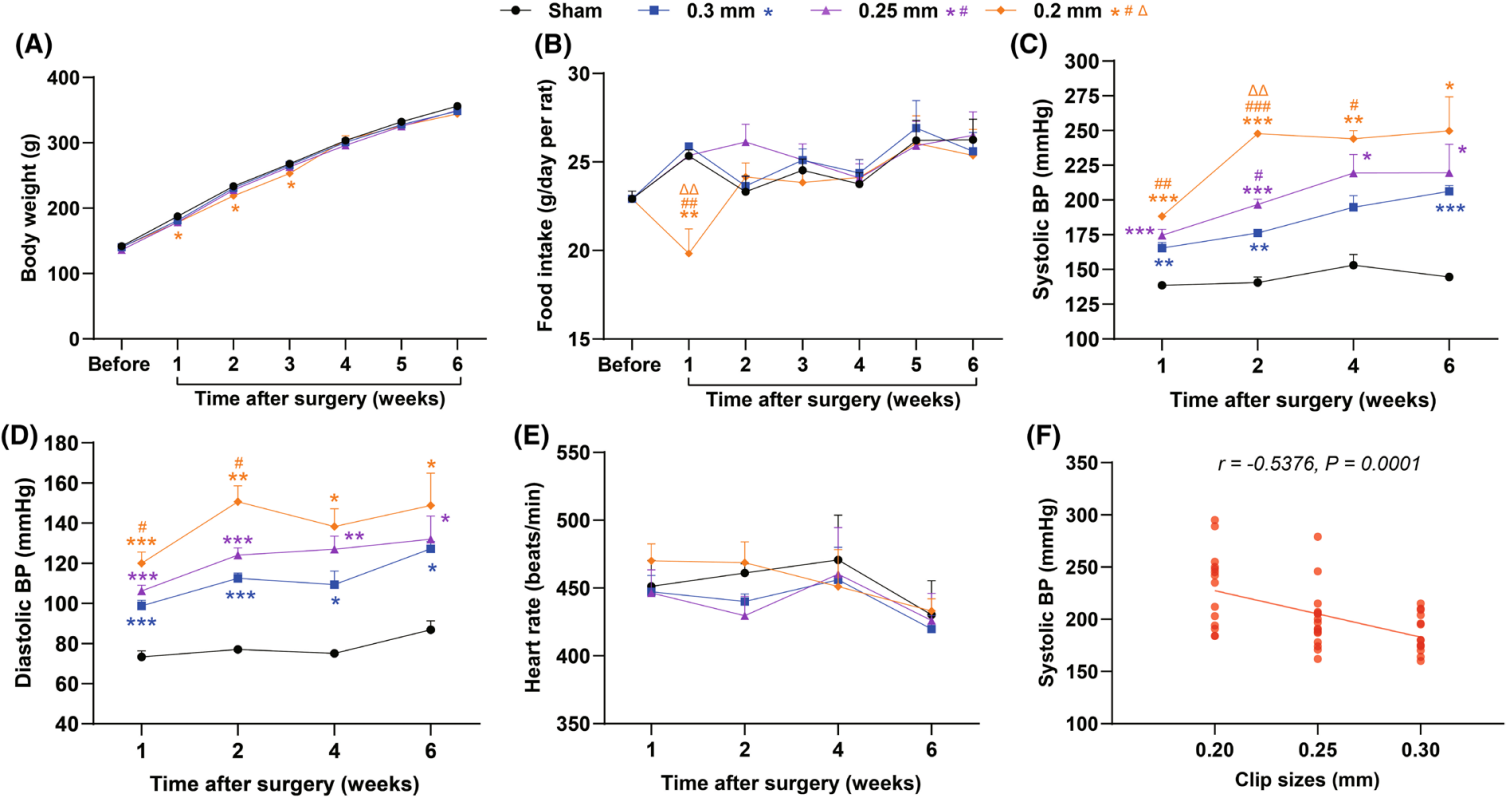
Changes in blood pressure (BP) and heart rate within 6 weeks after 2K2C surgery. (A) Body weight. (B) Food intake. (C) Systolic BP. (D) Diastolic BP. (E) Heart rate. (F) Correlation between systolic BP and clip sizes. Data are shown as the mean ± standard error mean (SEM). *n* = 3–6 animals per group. *p < 0.05, **p < 0.01, ***p < 0.001 versus sham. ^#^ *p* < 0.05, ^##^ *p* < 0.01, ^###^ *p* < 0.001 versus 0.3 mm. ^Δ^ *p* < 0.05, ^ΔΔ^ *p* < 0.01 versus 0.25 mm. Two‐way ANOVA followed by Dunnett's test is used. Pearson correlation coefficient is used in (F).

We prepared another batch of sham‐operated and 2K2C model rats (0.3, 0.25, and 0.2 mm), and performed this experiment twice. The body weight and food intake were monitored in the following 6 weeks (Figure 3A,B), and only rats in the 0.2 mm group showed a slight decrease in body weight within 3 weeks after 2K2C surgery, and their food intake decreased more within 1 week, but then they all returned to normal. This phenomenon may be attributed to the changes caused by severe renal artery stenosis in a short period, and the rats required some time to adapt.

At 1, 2, 4, and 6 weeks after the 2K2C surgery, a number of rats were randomly selected from each group for BP measurement. As shown in Figure 3C, at 1 week post operation, rats in the 0.3, 0.25, and 0.2 mm groups already had a significant increase in systolic BP (165, 175, and 188 mmHg, respectively) compared with the sham‐operated group (139 mmHg), which were similar to the BP levels during 10 h (the acute phase) after 2K2C surgery. In the following 2, 4, and 6 weeks, we observed that the systolic BP increased in all 2K2C groups and reached 206, 220, and 250 mmHg at the 6th week. We also performed comparisons among the 0.3, 0.25, and 0.2 mm groups. The systolic BP of 0.25 mm group was significantly higher than that of the 0.3 mm group at 2 weeks. The systolic BP of 0.2 mm group was significantly higher than that of the 0.3 mm group at 1, 2, and 4 weeks, and also higher than that of the 0.25 mm group at 2 weeks. BP measurements at different time points during the acute phase as well as during the next 6 weeks further indicate a 100% occurrence of hypertension at the day of 2K2C surgery and a progressive development of hypertension thereafter.

It was also observed that the 0.3 mm clip group exhibited a sustained increase in BP within 6 weeks after 2K2C surgery (206 mmHg at the 6th week), but the 0.25 mm clip group reached a plateau at the 4th week (219 mmHg), and the 0.2 mm clip group reached a plateau much earlier, at 2 weeks postoperatively (248 mmHg). This means that the smaller the silver clip size, not only the higher the BP of the rats, but also the earlier to reach a BP plateau.

Changes in diastolic BP were similar to those in systolic BP, and all the three 2K2C model groups showed no significant changes in heart rate compared with the sham‐operated group (Figure 3D,E). There existed a negative correlation between BP levels and the clip sizes (Figure 3F).

### 3.4 Gross morphology indicates multi‐organ damage as early as 1 or 2 weeks after 2K2C surgery

**FIGURE 4:**
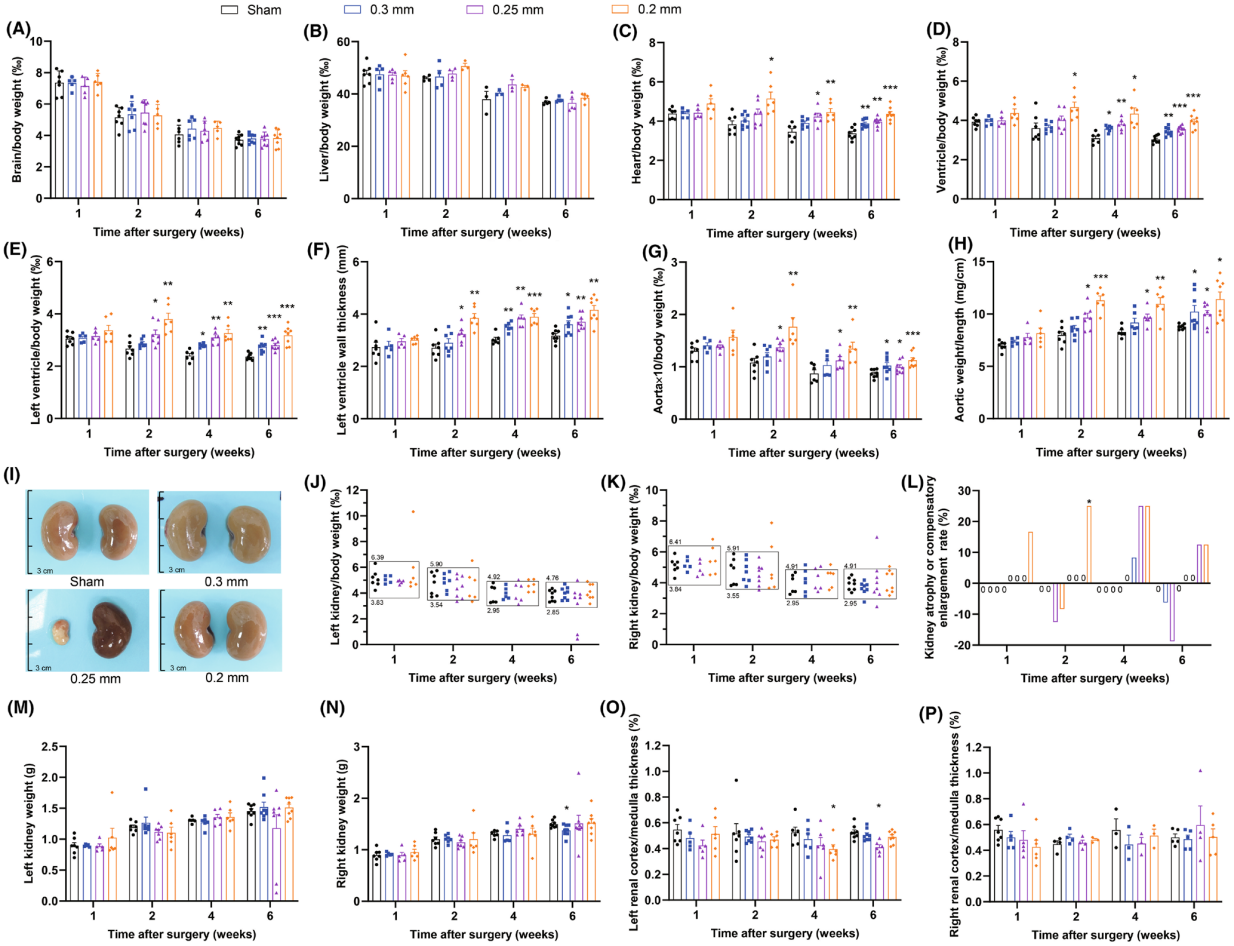
Organ gross morphology within 6 weeks after 2K2C surgery. (A) Brain/body weight. (B) Liver/body weight. (C) Heart/body weight. (D) Ventricle/body weight. (E) Left ventricle/body weight. (F) Left ventricle wall thickness. (G) Aorta × 10/body weight. (H) Aortic weight/length. (I) Representative images of kidney enlargement or atrophy in each group at 6th week. (J, K) Left and right kidney/body weight, with normal values shown in the boxes, and those above the boxes are enlarged and those below are atrophied. (L) Kidney atrophy or compensatory enlargement rate (based on the number of kidneys). (M) Left kidney weight. (N) Right kidney weight. (O) Left renal cortex/medulla thickness. (P) Right renal cortex/medulla thickness. Data are shown as the mean ± standard error mean (SEM). *n* = 4–8 animals per group. *p < 0.05, **p < 0.01, ***p < 0.001 versus sham. Two‐way analysis of variance (ANOVA) followed by Dunnett's test is used. Chi‐square test is used for (L).

Following BP measurements at the 1st, 2nd, 4th, and 6th weeks, rats were subjected to gross and histological morphology analysis. As shown in Figure 4, gross detection demonstrated little effects of 2K2C on the brain and liver (Figure 4A,B), but significant effects on the heart, aorta, and kidneys (Figure 4C–P).

At the 1st week, only the 0.2 mm group tended to cardiac and aortic hypertrophy, which was significant at the 2nd week. The 0.3 mm and 0.25 mm groups caused less organ damage than 0.2 mm group. As shown in Figure 4C–H, the relative weights of the heart, ventricle, left ventricle, and aorta, as well as the left ventricular wall thickness and aortic weight to length ratio, increased significantly, suggesting that the 2K2C models prepared with 0.3, 0.25, and 0.2 mm silver clips result in cardiac and aortic hypertrophy.

Renal atrophy or compensatory enlargement of some kidneys was observed (Figure 4I). The kidney weights of the sham‐operated group were used as reference, and their mean value ±25% was taken as the normal range. Kidney weights less than the low limit of normal range were defined as atrophy, and more than the high limit of normal range were defined as compensatory enlargement. Results showed that renal compensatory enlargement occurred in the 0.2 mm clip group as early as 1 week after 2K2C surgery. From 2 weeks postoperatively, some rats also experienced renal atrophy (Figure 4J,K). The onset time of renal atrophy or compensatory enlargement was clip size‐dependent, the earlier, smaller the size (Table 1). Unilateral renal atrophy, unilateral renal compensatory enlargement, bilateral renal atrophy, bilateral renal compensatory enlargement, and bilateral changes with one side of renal atrophy and another side of renal compensatory enlargement were observed in different 2K2C rats. The probability of atrophy or compensatory enlargement of the kidneys gradually increased with time and decreasing size of the silver clips (Figure 4L; Table 1). The incidence rate was low (3.8%) in 0.3 mm clip group without statistical significance, but significantly increased to 18.5% and 21.2%, respectively, in 0.25 mm and 0.2 mm clip groups. The bilateral incidence rate also significantly increased to 14.8% and 11.5%, respectively, in 0.25 and 0.2 mm clip groups (Table 1). However, the degree of renal atrophy or enlargement was not well correlated with clip sizes, not the smaller the clip, the more severe the atrophy or enlargement. We observed that the 0.25 mm group had severe renal atrophy in two rats (shown in Figure 4I,J,M). The weights of the left and right kidneys in most of 2K2C groups seemed to have no significant changes compared with the corresponding sham groups (with one significant change demonstrating a reduction in kidney weight), which may be related to renal atrophy or compensation (Figure 4M,N). The left and right renal cortex to medulla thickness ratios in most of 2K2C groups appeared to have no significant changes compared with the corresponding sham groups (with two significant changes demonstrating a reduction in the ratios) (Figure 4O,P).

**TABLE 1:** Renal atrophy or compensatory enlargement in two‐kidney two‐clip (2K2C) rats.

	Clip size (mm)	Onset time	Incidence rate	Bilateral incidence rate
Sham	/	/	0 (0/56)	0 (0/56)
2K2C	0.3	4 weeks after 2K2C	3.8% (2/52)	0 (0/52)
2K2C	0.25	2 weeks after 2K2C	18.5% (10/54)***	14.8% (8/54)**
2K2C	0.2	1 weeks after 2K2C	21.2% (11/52)***	11.5% (6/52)**

*Note*: Data based on the number of kidneys.

** *p* < 0.01.

*** *p* < 0.001 versus Sham.

Together, these gross detections demonstrate cardiac and aortic hypertrophy as well as renal atrophy and compensatory enlargement in 2K2C rats that can be examined as early as 1 or 2 weeks after 2K2C operation.

### 3.5 Histological morphology indicates multi‐organ damage as early as 1 or 2 weeks after 2K2C surgery

**FIGURE 5:**
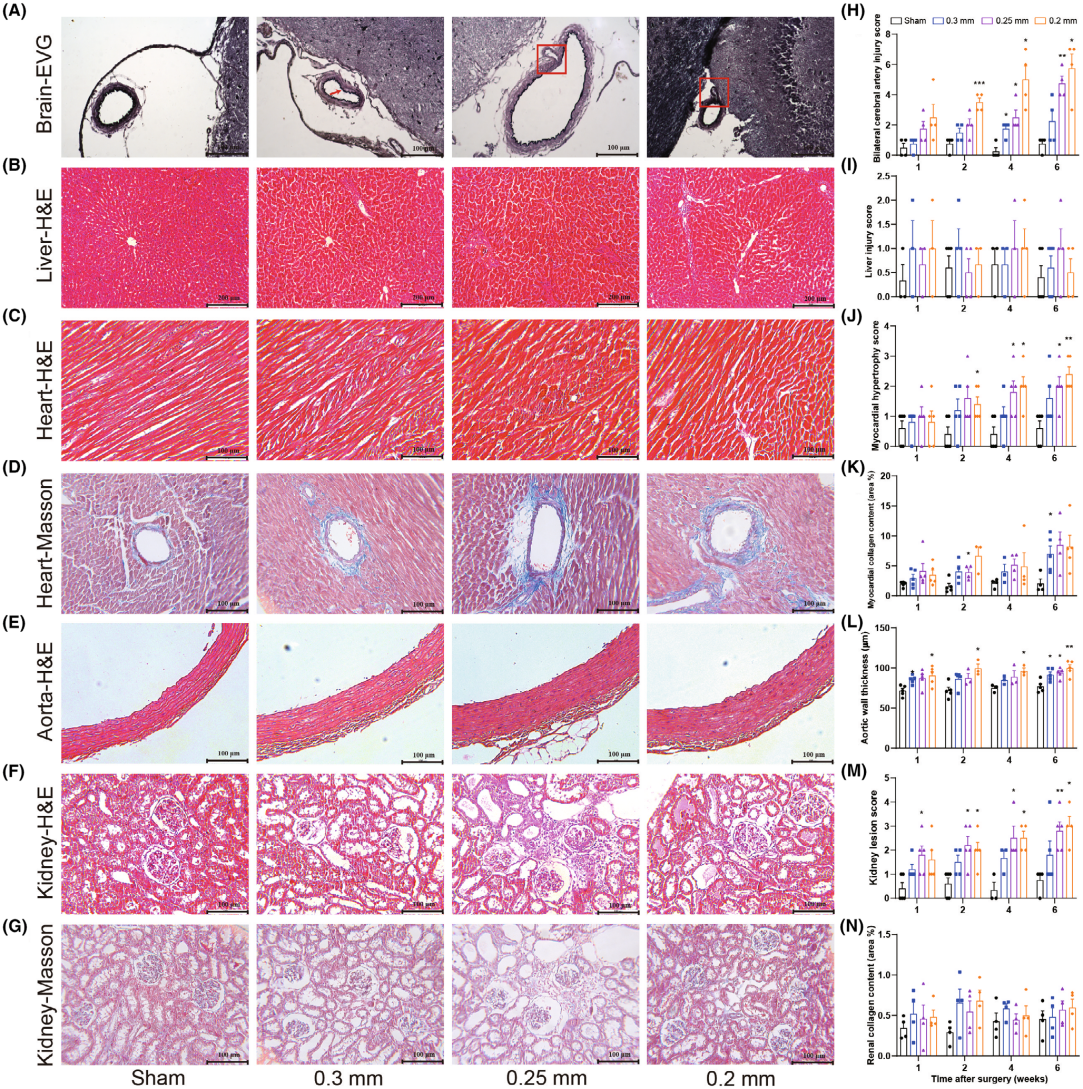
Changes in organ histological morphology after 2K2C surgery. (A–G) Representative images in week 6 of brain Verhoeff's Van Gieson (EVG) staining, Magnification: 200X (eyepiece 10X, objective lens 20X) (A), liver hematoxylin–eosin (H&E) staining, Magnification: 100x (eyepiece 10x, objective lens 10x) (B), heart H&E staining, Magnification: 200X (eyepiece 10X, objective lens 20X) (C), heart Masson staining, Magnification: 200X (eyepiece 10X, objective lens 20X) (D), aorta H&E staining, Magnification: 200X (eyepiece 10X, objective lens 20X) (E), kidney H&E staining, Magnification: 200X (eyepiece 10X, objective lens 20X) (F), and kidney Masson staining, Magnification: 200X (eyepiece 10X, objective lens 20X) (G). (H) Bilateral cerebral artery injury score. (I) Liver injury score. (J) Myocardial hypertrophy score. (K) Myocardial collagen area fraction. (L) Aortic wall thickness. (M) Kidney lesion score. (N) Renal collagen area fraction. Data are shown as the mean ± standard error mean (SEM). *n* = 3–5 animals per group. *p < 0.05, **p < 0.01, ***p < 0.001 versus sham. Two‐way analysis of variance (ANOVA) followed by Dunnett's test is used. [Correction added on 21 Aug 2025 after first online publication: The magnification level has been added in the figure 5 caption.]

Although there were no significant changes in the relative brain weights (Figure 4A), EVG staining of cerebral vessels displayed progressive and stenosis‐degree‐dependent cerebrovascular injury (vascular wall elastic fiber thinning, breakage, deformation, etc.) after 2K2C surgery (Figure 5A,H; Figures S1–S3A). Similarly there were no significant changes in the relative liver weights; H&E staining; H&E staining of the liver exhibited less damage with no significant differences among groups (Figure 5B,I; Figures S1B–S3B).

H&E staining of the heart showed progressively myocardial hypertrophy (cardiomyocytes became wider) against hypertension time (Figure 5C,J; Figures S1–S3C). Masson staining indicated collagen fibers in all groups of myocardial tissue, with a significant increase or increased tendency in the three groups of 2K2C rats, suggesting cardiac fibrosis (Figure 5D,K). It was noted that intramyocardial vascular lesions occurred (Figure 5D; Figures S1–S3D). Measurements of the aortic wall thickness also showed progressive and stenosis‐degree‐dependent thickening from 1 week after 2K2C surgery (Figure 5E,L; Figures S1–S3E).

H&E staining of the kidney showed glomerular atrophy, interstitial enlargement, inflammatory cell infiltration, and formation of cavities, especially in the 0.25 mm and 0.2 mm groups (Figure 5F,M, Figures S1–S3F). Masson staining suggested that the kidney lesions did not correlate well with fibrosis (Figure 5G,N; Figures S1–S3G).

Together, histological analysis further supports multi‐organ damage in 2K2C rats, mainly including cerebrovascular and cardiovascular lesions, cardiac and aortic hypertrophy, as well as renal atrophy and compensatory enlargement that can be examined as early as 1 or 2 weeks after 2K2C operation.

### 3.6 Routine serum biochemistry indicates multi‐organ dysfunction in 2K2C rats with AST as an early biomarker for severe hypertension‐related organ damage

**FIGURE 6:**
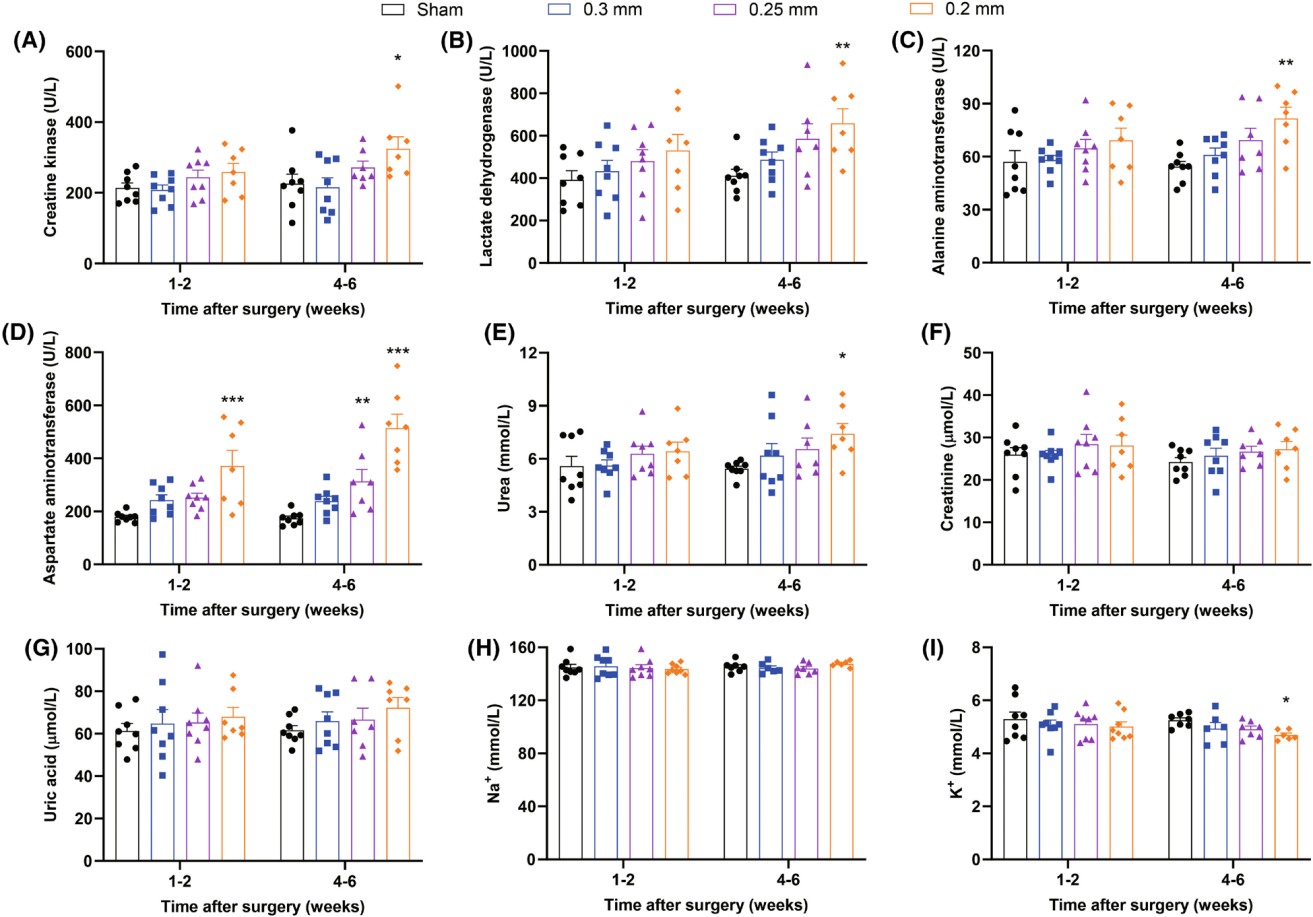
Changes in serum biochemical indices after 2K2C surgery. (A) Serum creatine kinase. (B) Serum lactate dehydrogenase. (C) Serum alanine aminotransferase. (D) Serum aspartate aminotransferase. (E) Serum urea. (F) Serum creatinine. (G) Serum uric acid. (H) Serum Na^+^. (I) Serum K^+^. Data are shown as the mean ± standard error mean (SEM). *n* = 7–8 animals per group. *p < 0.05, **p < 0.01, ***p < 0.001 versus sham. Two‐way analysis of variance (ANOVA) followed by Dunnett's test is used.

As shown in Figure 6A–I, serum biochemical assays showed mild effects of 2K2C in each group at 1–2 weeks, but 2K2C model prepared by 0.2 mm silver clips showed increased CK, LDH, ALT, AST, urea levels (Figure 6A–E), and decreased serum K^+^ level at 4–6 weeks after 2K2C surgery (Figure 6I), indicating myocardial and hepatic injury and renal dysfunction, whereas 0.3 mm and 0.25 mm clip groups were still less affected in the biochemical indicators. Specifically, among nine examined parameters, AST was a relatively sensitive parameter reflecting an only early change at 1–2 weeks (in 0.2 mm clip group) as well as an only change in the 0.25 clip group (at 4–6 weeks). These results suggest the value of these non‐invasive routine serum biochemical assays in evaluating organ damage and dysfunction of severe hypertension.

## 4 DISCUSSION

**FIGURE 7:**
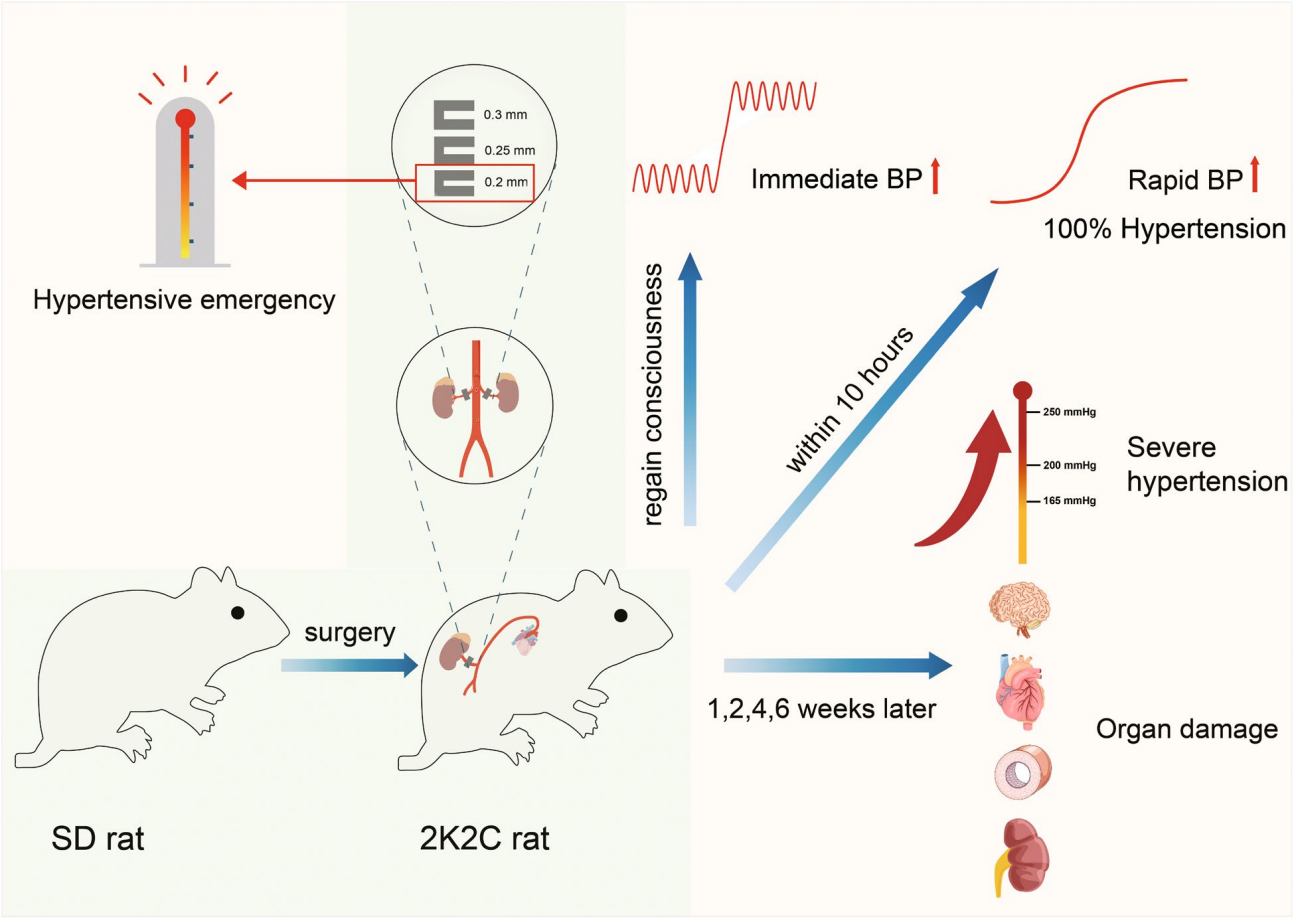
Characteristics of the 2K2C rat model. Blood pressure increases immediately when the 2K2C rats (made with 0.3, 0.25, and 0.2 mm U‐shaped clips) regain consciousness from anesthesia, and 100% of the rats develop hypertension on the day of 2K2C surgery. Then the blood pressure continues to rise, resulting in severe hypertension and organ damage to the brain, heart, aorta, and kidneys at 1, 2, 4, and 6 weeks after 2K2C surgery. The 2K2C rat model made with 0.2 mm clips is also suitable for hypertensive emergency research.

The 2K2C rat model can lead to severe hypertension and organ damage and is similar to the characteristics of renovascular hypertension in humans.^27^ Our study shows that the systolic BP increases immediately after awakening from 2K2C surgery. Systolic BP levels at 10 h and 1 week after surgery demonstrate that even the lowest systolic BP in the 0.3 mm clip group exceeds 160 mmHg. Systolic BP reaches a plateau after 2 weeks and is as high as 248 mmHg in the 0.2 mm clip group. The incidence of hypertension produced by all three sizes of clips is 100%, which can be detected on the day of 2K2C surgery and thereafter. Morphological and pathological analyses also show that multi‐organ damage has already occurred at 1 or 2 weeks postoperatively (Figure 7). Serum biochemical tests indicate that the persistent high BP levels can cause multi‐organ dysfunction. These changes appear correlated with the degree of renal artery stenosis (produced by different inner diameters of 0.3, 0.25, and 0.2 mm clips, which can reduce renal blood flow at different degrees), with the higher the degree of stenosis, the more severe the hypertension and organ damage.

In previous studies, it is unknown when BP starts to rise, and after 2K2C surgery, it usually needs 2–4 weeks to raise BP before medication is administered.^11^, ^12^, ^13^, ^14^, ^28^, ^29^ Our study first shows that after 2K2C surgery, BP increases immediately when the rats regain consciousness, without a long time waiting. However, BP does not elevate when placing silver clips to narrow the renal arteries, whether it is a 0.3, 0.25, or 0.2 mm silver clip. Theoretically, reduced renal blood flow activates RAAS, which promotes renin release and then increases angiotensin II (Ang II) level, constricting blood vessels and raising BP,^7^, ^30^, ^31^ but BP does not increase until the rats are awake. We speculate that this may be due to the inhibition of renin release under anesthesia. It is known that isoflurane anesthesia mainly depresses the central nervous system,^32^ and renin secretion is innervated by sympathetic nerves^33^, ^34^; thus the renin may not be released without the RAAS activation during the operation under anesthesia. We also observed an elevation of BP in sham‐operated rats when they become conscious, which may be due to the animals being de‐anesthetized and pain contingency. We compared BP in 2K2C rats with the sham operated animals and confirmed that BP increases immediately after consciousness from 2K2C operation. The subsequent BP measurements at 1, 2, 4, and 6 weeks suggest that the BP progressively increases with a decrease in clip size and the extension of the modeling time. It appears that the smaller the silver clip size, the faster the BP of the rats reaches the plateau, such as the 0.2 mm clips for 2 weeks (248 mmHg), 0.25 mm clips for 4 weeks (219 mmHg), and 0.3 mm clips perhaps not reaching a plateau at 6 weeks of observation time (206 mmHg). There are some disadvantages in the present study. Although we speculated that the change in renin levels led to the increase in BP, we did not measure it. In our previous study, we measured the serum aldosterone at 10 weeks after 2K2C surgery, which was significantly higher than that of sham group, indicating that the RAAS system was activated.^11^ Other studies also found that 2K2C rats showed an increase in BP and serum Ang II at the 10th week.^35^ In 2K1C rats, plasma and renal Ang II increased at 7 days after surgery.^36^ These results suggest that the RAAS system is activated after renal artery stenosis in both 2K1C and 2K2C rats. The work of Bencze et al. also enlightens our understanding of the mechanism research, which used captopril, pentolinium, and l‐nitroarginine methyl ester (L‐NAME) to study BP responses by sequentially blocking the RAAS, sympathetic nervous system (SNS), and nitric oxide (NO) synthesis.^37^ In the future study, we will test the levels of RAAS parameters such as renin and Ang II in serum, kidney, and cardiovascular organ, and observe the BP responses by blockade of certain BP‐related regulations to know the underlying mechanism.

In addition to severe hypertension, 2K2C rats exhibit a progressive multi‐organ damage. First, cerebrovascular lesions are obvious at 2 weeks after 2K2C operation and develop progressively with time prolongation. In the brain EVG staining, the fracture and deformation of cerebral vascular elastic fibers are consistent with the cerebral small vessel injury caused by hypertension, which can lead to cognitive dysfunction.^38^, ^39^ In fact, our most recent study has demonstrated cognitive dysfunction in 2K2C rats.^12^ Second, the heart in the 0.2 mm clip group tends to hypertrophy at 1 week after 2K2C surgery, cardiac hypertrophy is obvious at 2 weeks and in progress thereafter. Different sizes of clips gradually increase cardiac and myocardial hypertrophy with size reduction and time prolongation. A progress lesion also occurs in the intramyocardial arteries. Third, aortic hypertrophy occurs as early as 1 week after 2K2C operation and displays progress with time. Finally, renal lesions occur as early as 1 week after 2K2C operation and display progress with time. Our study shows that the 2K2C model can cause rapid organ damage, providing a more definitive time point of injury for hypertension research.

Several routine serum biochemical parameters are used for measurements of cardiac enzymes, liver function, and renal function in the present study. Changes in serum biochemical parameters occur mainly at the stage of severe organ damage (0.2 mm clips, 4–6 weeks after 2K2C surgery). For cardiac enzymes, we have measured CK and LDH. CK is present in significant concentrations in skeletal muscle and cardiac muscle.^40^ LDH is widely distributed in many organs and tissues, with the highest levels found in the heart, liver, spleen, lungs, kidneys, pancreas, brain, bones, and others. Significant elevations of CK and LDH at 4–6 weeks suggest myocardial damage and release of cardiac enzymes into the bloodstream. Also, other organ damages such as brain and kidney damage might contribute to LDH increase. For liver function, AST increases to a higher degree than ALT, which is usually a severe liver injury, but H&E staining shows no serious lesions in the liver. One explanation is that because ALT and AST are also present in cardiomyocytes and because AST is predominantly distributed in the myocardium, followed by liver, skeletal muscle, and kidney,^41^ AST elevation in the present study may be related to cardiac injury and/or other organ damage. For renal function, we measured serum creatinine, urea, uric acid, Na^+^, and K^+^, and there is only one increase in urea level with no other obvious deteriorations due to the strong compensatory function of the kidney. Perhaps the renal function deteriorations need a longer period, or other indicators in the urine may be more sensitive reflecting renal damage. In the future study, metabolic cages will be used to detect food and water consumption, as well as urine volume, protein, and others, so as to more reasonably explain the pathological changes in kidneys. Among all examined nine parameters, AST appears as an early biomarker for indication of severe hypertension‐related organ damage. In clinical settings, direct tissue examination is difficult to perform, and blood biochemical tests are easy to perform. Therefore, it is meaningful to identify early biomarkers for hypertensive organ damage. Future clinical study may pay attention to AST for its importance in hypertensive organ damage, especially in severe hypertension and hypertensive emergencies.

The clinical hypertensive emergencies are defined as a sudden and significant increase in BP (usually systolic BP >180 mmHg and/or diastolic BP >120 mmHg) in patients with primary or secondary hypertension, accompanied by hypertension‐related target organ damage or progressive aggravation of the original functional impairment of the organs.^1^, ^3^, ^42^ The main features are a sudden increase in BP and a progressive organ damage. In the present study, 2K2C rats made with 0.2 mm silver clips display a rapid increase in BP with approximately 186 mmHg at 10 h postoperatively and maintain this BP level at 1 week after surgery (approximately 188 mmHg). In subsequent BP and organ damage measurements at 1, 2, 4, and 6 weeks after 2K2C surgery, rats in the 0.2 mm clip group rapidly reach a BP plateau of 248 mmHg at 2 weeks postoperatively, with a progressive cerebrovascular, cardiac, aortic, and renal damage. These characteristics in 0.2 mm clip 2K2C rats are consistent with the features of hypertensive emergencies. Therefore, we propose that the 2K2C rat model made by 0.2 mm silver clips can be used as a model of hypertensive emergencies. Only male rats have been used in the present study because females are susceptible to reproductive and hormonal disturbances that result in less stable BP levels.^43^, ^44^, ^45^ It is reasonable that hypertensive emergency also applies to female rats, as they are all affected by narrowing of the renal arteries and reduced blood flow. However, this extension remains to be verified in a new study using female rats.

## 5 CONCLUSIONS

In summary, as shown in Figure 7, the present study uses 0.3, 0.25, and 0.2 mm silver clips to prepare severe renovascular hypertension of 2K2C rats, revealing for the first time that BP elevation occurs when the rats are awake from 2K2C surgery, and that the BP can reach a high level (>160 mmHg) 10 h after 2K2C surgery, with a 100% incidence of hypertension at the day of operation. Long‐term measurements show a rapid increase in BP after 2K2C surgery, reaching a very high plateau after 2 weeks (0.2 mm clips, 248 mmHg) and 4 weeks (0.25 mm, 219 mmHg), and a stenosis‐effect relationship among the 0.3, 0.25, and 0.2 mm clips for all examined time points (1, 2, 4, and 6 weeks), with BPs of 206, 220, and 250 mmHg, respectively, at 6 weeks after 2K2C surgery. Morphological measurements suggest that progressive organ damage can occur as early as 1 or 2 weeks after 2K2C surgery. Routine serum biochemical changes can indicate severe organ damage and/or dysfunction in 2K2C rats. Among them, AST is of particular note, and it may be an early biomarker for severe hypertension‐related organ damage, deserving further verification in clinical settings. We also propose that the 2K2C rats prepared with 0.2 mm silver clips can be used as a model for hypertensive emergencies. All these findings are valuable to better understand the characteristics of bilateral renovascular hypertension and are useful for antihypertensive drug development and treatment.

## AUTHOR CONTRIBUTIONS

**Jia‐Sheng Tian:** Data curation; formal analysis; methodology; writing – original draft. **Qi‐Sheng Ling:** Data curation; formal analysis; methodology; validation. **Yu‐Chen Wei:** Data curation; formal analysis; methodology. **Dao‐Xin Wang:** Data curation; formal analysis; methodology. **Chao‐Yu Miao:** Conceptualization; formal analysis; funding acquisition; methodology; resources; writing – review and editing.

## FUNDING INFORMATION

This work was supported by grants from the National Natural Science Foundation of China Major Projects (nos.: 82030110, 82330117, and 81730098 to Chao‐Yu Miao).

## CONFLICT OF INTEREST STATEMENT

The authors declare no conflicts of interest.

## ETHICS STATEMENT

Animal experiments were approved by the Committee on Ethics of Medical Research, Second Military Medical University / Naval Medical University.

## Supporting information


Figures S1–S3.
