# Supplementary material for: Rapid severe hypertension and organ damage in two‐kidney two‐clip rats produced by different sizes of clips
Source: Animal Model Exp Med. 2025 May 14;8(8):1456–67. doi: 10.1002/ame2.70027 (PMC12464872; doi:10.1002/ame2.70027)
Supplement: Supplementary file 1 — Figures S1–S3. [file AME2-8-1456-s001.docx]

**Supplementary Materials**

**Title: Rapid severe hypertension and organ damage in two-kidney two-clip rats produced by different sizes of clips**

**The supplementary materials include:**

**Representative pictures of organ histological morphology at 1, 2, and 4 weeks after 2K2C surgery (Fig. S1, Fig. S2 and Fig. S3)**


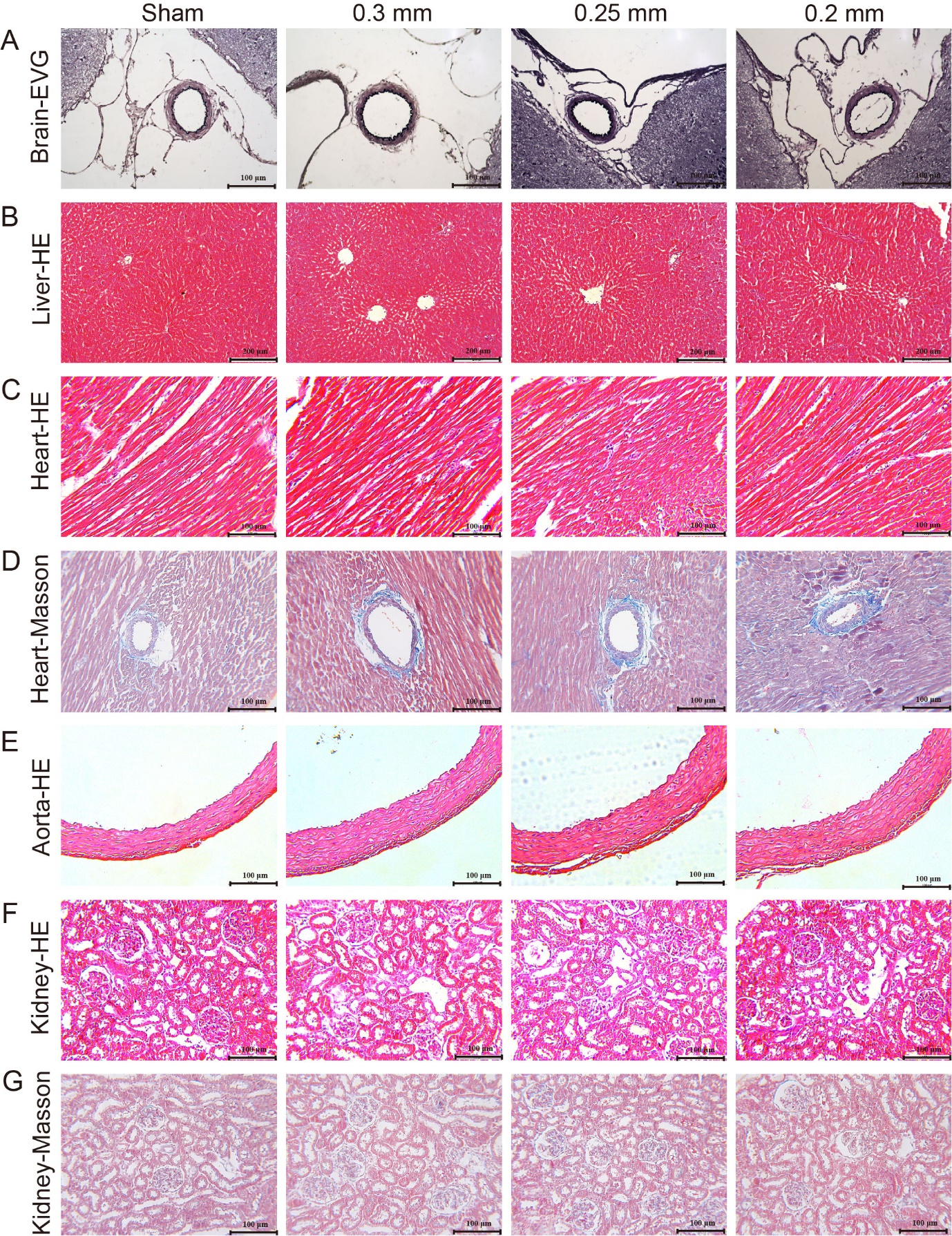


**Fig. S1** Changes in organ histological morphology at 1 week after 2K2C surgery using internal diameter of 0.3 mm, 0.25 mm and 0.2 mm clips. Representative pictures of brain EVG staining (A), liver H&E staining (B), heart H&E staining (C), heart Masson staining (D), aorta H&E staining (E), kidney H&E staining (F), and kidney Masson staining (G).


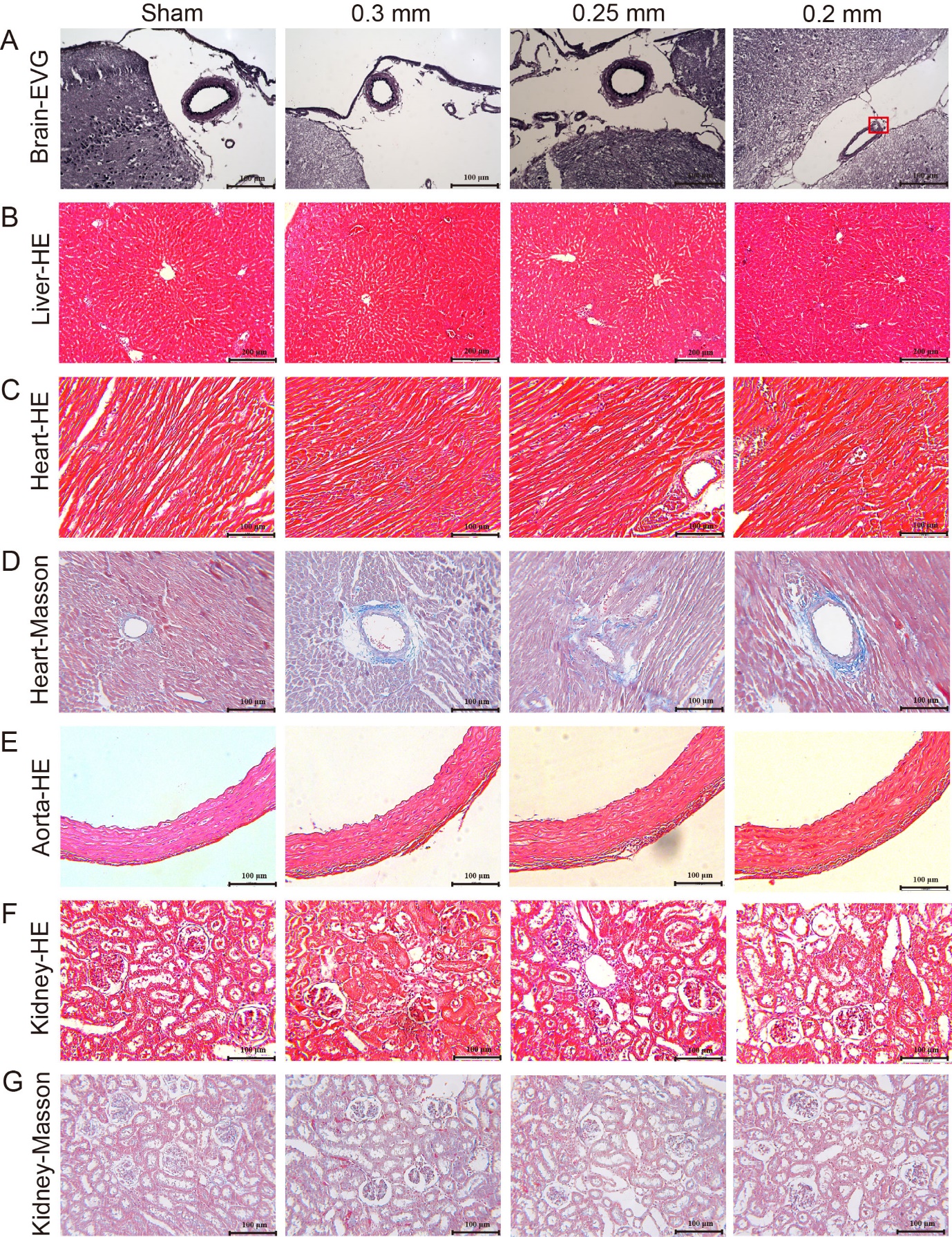


**Fig. S2** Changes in organ histological morphology at 2 weeks after 2K2C surgery using internal diameter of 0.3 mm, 0.25 mm and 0.2 mm clips. Representative pictures of brain EVG staining (A), liver H&E staining (B), heart H&E staining (C), heart Masson staining (D), aorta H&E staining (E), kidney H&E staining (F), and kidney Masson staining (G).


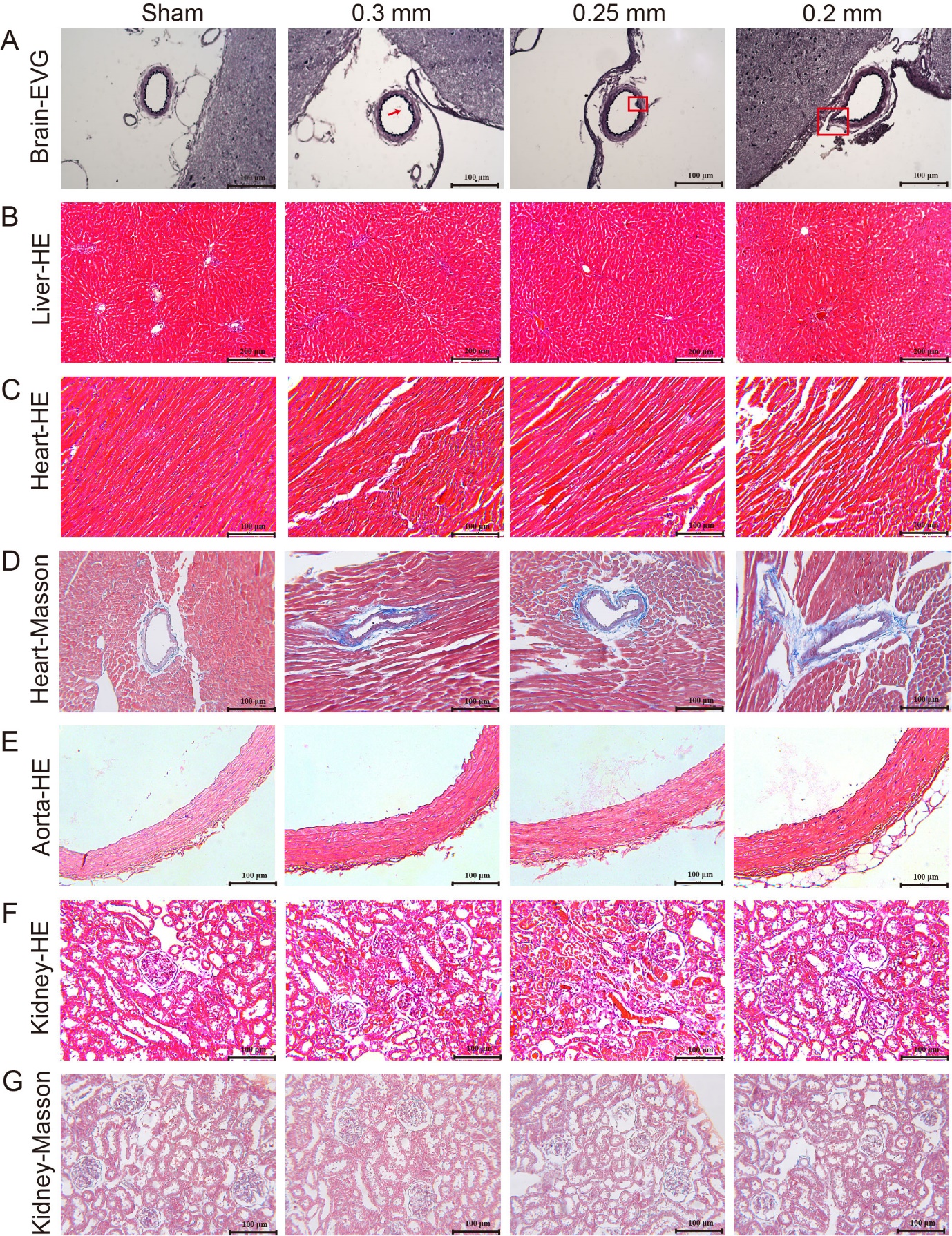


**Fig. S3** Changes in organ histological morphology at 4 weeks after 2K2C surgery using internal diameter of 0.3 mm, 0.25 mm and 0.2 mm clips. Representative pictures of brain EVG staining (A), liver H&E staining (B), heart H&E staining (C), heart Masson staining (D), aorta H&E staining (E), kidney H&E staining (F), and kidney Masson staining (G).
